# The impact of long-term changes in metabolic status on cardiovascular biomarkers and microvascular endothelial function in middle-aged men: a 25-year prospective study

**DOI:** 10.1186/s13098-015-0074-8

**Published:** 2015-09-17

**Authors:** Magdalena Kwaśniewska, Joanna Kozińska, Elżbieta Dziankowska-Zaborszczyk, Tomasz Kostka, Anna Jegier, Ewa Rębowska, Milena Orczykowska, Joanna Leszczyńska, Wojciech Drygas

**Affiliations:** Department of Preventive Medicine, Medical University of Lodz, Zeligowskiego 7/9, 90-752 Lodz, Poland; Department of Epidemiology and Biostatistics, Medical University of Lodz, Zeligowskiego 7/9, 90-752 Lodz, Poland; Department of Geriatrics, Medical University of Lodz, Zeromskiego 113, Lodz, Poland; Department of Sports Medicine, Medical University of Lodz, Pomorska 251, 92-213 Lodz, Poland; Central Clinical Hospital, Medical University of Lodz, Pomorska 251, 92-213 Lodz, Poland; Department of Epidemiology, Cardiovascular Disease Prevention and Health Promotion, Institute of Cardiology, Niemodlinska 33, 04-635 Warsaw, Poland

**Keywords:** Metabolic syndrome, Disorders, Biomarkers, Endothelial, Reactive hyperemia, Physical activity

## Abstract

**Background:**

The aim of this analysis was to examine long-term effects of changes in metabolic status on microvascular endothelial function and cardiovascular diseases (CVD) biomarkers among physically active middle-aged men.

**Methods:**

Metabolically healthy men (n = 101, mean age 59.7 years), free of symptoms and treatment, have been prospectively observed for their lifestyle and CVD risk factors (observation period 24.7 years). At the latest follow-up (2011/2012) a set of CVD biomarkers was measured using enzyme-linked immunosorbent assay. Microvascular endothelial function was evaluated by means of the reactive hyperemia index (RHI) using Endo-PAT2000 system. At follow-up the participants were divided into metabolically healthy (0–1 metabolic parameters) and metabolically unhealthy (≥2 metabolic parameters) groups. Metabolic syndrome was defined according to the NCEP ATP III definition.

**Results:**

Traditional metabolic risk factors were significantly associated with hsCRP, ox-LDL, Il-6, leptin and adiponectin/leptin ratio. Reactive hyperemia index was negatively related to body mass (p < 0.01), waist circumference (p < 0.05), triglycerides (p < 0.01), TG/HDL ratio (p < 0.01), uric acid (p < 0.05), sICAM-1 (p < 0.05) and Il-6 (p < 0.05), and positively to HDL-C (p < 0.01) and leisure-time physical activity (p < 0.01). Men who maintained metabolically healthy status (n = 47) through the observation had significantly lower hsCRP and uric acid (p < 0.05), higher adiponectin/leptin ratio (p < 0.05), higher mean RHI and lower prevalence of endothelial dysfunction (p < 0.05) as compared to the metabolically unhealthy group (n = 54). Regular physical activity level was significantly higher among metabolically healthy individuals during the whole observation.

**Conclusions:**

Even subtle changes in metabolic profile influence inflammatory biomarkers and microvascular endothelial function. Leptin, adiponectin/leptin ratio and hsCRP are significant predictors of metabolic profile. Interleukine-6 and sICAM-1 may be used as indicators of early endothelial dysfunction in asymptomatic men. High leisure-time physical activity level is an important contributor of metabolically healthy profile through middle adulthood.

## Background

Metabolic disorders, including visceral obesity, elevated blood pressure, lipids and glucose abnormalities, are known to be associated with cardiovascular diseases (CVD) [1]. Increased visceral fat and other metabolic disturbances may impair nitric oxide bioactivity and cause endothelial dysfunction independently of traditional CVD risk factors [2]. It has been also shown that some metabolic components may enhance the release of several hormones, inflammatory cytokines and molecules what may results in an impairment of endothelial function [3–6]. Identification of key biomarkers could be an important tool for early detection of metabolic risk before the appearance of its frank components.

Endothelial function measurement is thought to be a sensitive marker for a variety of cardiovascular (CVD) conditions and vascular health [7]. As endothelial dysfunction is the initial perturbation in development of atherosclerosis, identifying individuals with impaired endothelial function may substantially influence adverse CVD events [8, 9].

Physical activity (PA) is one of the fundamental factors modifying the metabolic profile and provides substantial CVD benefits [10–13]. It is postulated that protective effects of PA on vascular system may result from affecting some biomarkers involved in development of atherosclerosis and endothelial function, although precise mechanisms of this relationship remain unclear [14, 15].

Therefore, the aim of this analysis was to examine long-term effects of changes in metabolic status on endothelial function and CVD biomarkers in the cohort of physically active men. In the Healthy Men Clinic of the Medical University of Lodz (Poland) we have a unique opportunity to follow-up the population of apparently healthy physically active men for a period of above 25 years.

## Methods

### Study design and subjects

All the subjects were provided with written information about the purpose and methodology of the study. The protocol of the project has been approved by the Medical University of Lodz Ethics Committee.

Recruitment procedure and other methods has been described in our previous paper [12]. Briefly, the subjects of the study consisted of male volunteers who regularly attended the Healthy Men Clinic and the Department of Preventive Medicine, Medical University of Lodz (Poland) since 1985. Subjects were considered to be eligible if before the examination they were asymptomatic, free from chronic diseases and treatment (including aspirin, statins and anti-hypertensive agents) and any important disability or dementia.

Previous structured follow-up examination was realized in 2003–2005 within a scientific grant of the Ministry of Science and Higher Education. Data gathered in 2003–2005 were assessed for eligibility and 193 subjects met preliminary inclusion criteria. Of the 193 subjects personally invited by mail, 10 men did not respond, 3 died in the years 2003–2012 (1 stroke, 1 cancer, 1 car accident), 23 were diagnosed with chronic diseases and 24 was taking CVD drugs (information collected during a phone call). Of the 134 men (mean age 62.1 ± 3.6 years) who attended the Clinic in 2011/12, we disqualified 33 subjects due to abnormalities found during physical examination, echocardiography or exercise test, and/or taking agents modifying CVD risk.

Therefore, the final cohort of the latest follow-up (2011–2012) consisted of 101 asymptomatic men aged 50–77 years (mean age 59.7 ± 9.0 years; mean observation period: 24.7 ± 4.1 years). The subjects were white men, predominantly married, white collar workers with university or secondary educational level whose occupational activity was low. In the years 1985–2005 all subjects participated in a similar panel of procedures including a detailed interviewer-administered questionnaire, anthropometric and biochemical measurements, resting electrocardiogram and the graded submaximal exercise test. The latest follow-up (2011/2012) included also an assessment of atherosclerosis indices and novel biomarkers.

### Metabolic risk factors and physical activity measures

All the participants have completed a medical history questionnaire which included information on their health status, family history of CHD, smoking, nutrition habits, alcohol consumption and detailed information about leisure-time physical activity (LTPA) since the initial visit.

Fasting blood samples were drawn from the antecubital vein. Enzymatic methods were used to determine serum total cholesterol, glucose, triglycerides, uric acid concentrations (COBAS INTEGRA 400 Plus, Roche). High-density lipoproteins (HDL-C) were measured by the precipitation method. Concentration of low-density lipoproteins (LDL-C) was estimated using the Friedewald formula. Anthropometric data were collected by standard methods.

Metabolic disorders for males were defined according to the National Cholesterol Education Program Adult Treatment Panel III (NCEP ATP III) guidelines [16]. Metabolic syndrome was defined as the presence of 3 or more of the following parameters: waist circumference (WC) ≥102 cm, systolic and/or diastolic blood pressure (BP) ≥130/85 mmHg, triglycerides (TG) ≥1.7 mmol/l, HDL-C <1.0 mmol/l, fasting plasma glucose (FPG) ≥6.1 mmol/l. [16]. Individuals with zero or one metabolic disorder were categorized as metabolically healthy.

Leisure-time physical activity has been assessed by an interviewer-administered questionnaire. Exercise-related energy expenditure (EE) was assessed on the basis of the amount of hours earmarked for weekly recreational sport activities (kcal/week) according to the tables of Fox [17]. Historical LTPA was assessed according to Kriska [18] for the following periods of life: 12–34, 35–49, over 50 years old period, for the last 5 and 10 years and for the whole period from 12th year of life to the day of examination. All the measures of historical LTPA are expressed as hours per year for a given period.

In order to assess aerobic fitness the graded submaximal exercise test was carried out on a Monark type 818E (Stockholm, Sweden) bicycle ergometer with 30 W increments every 3 min to achieve at least 85 % of maximal age-predicted HR (220-age). The resultant linear regression equation was used to calculate the aerobic capacity index, i.e. physical working capacity at 85 % of the maximal heart rate (PWC85%HRmax) [19]. PWC85%HRmax was expressed as relative to body mass [PWC/kg (W kg^−1^)].

*Cardiovascular biomarkers.* Plasma concentration of high-sensitivity C-reactive protein (hsCRP), homocysteine (Hcy), interleukine-6 (Il-6), oxidized low-density lipoproteins (ox-LDL), soluble intracellular adhesion molecule-1 (sICAM-1), soluble vascular cell adhesion molecule-1 (VCAM-1), leptin, resistin, adiponectin and irisin were measured using enzyme-linked immunosorbent assay kit (Diaclone, France; Labor Diagnostika Nord, Germany; BioVendor, Czech Republic; Immundiagnostik AG, Germany; Axis-Shield Diagnostics Ltd., Scotland). Fasting serum was stored according to the manufacturer’s recommendations.

*Microvascular endothelial function.* Peripheral arterial tonometry signals were obtained using the EndoPAT 2000 device (Itamar MedicalInc., Caesarea, Israel) in participants resting in the supine position in quiet, temperature-controlled environment after an overnight fast. Subjects were also instructed to refrain from smoking and strenuous exercise at least 12 h before the examination. Briefly, a PAT finger probe was placed on each index finger. Pulsatile volume changes of the distal digit induced pressure alterations in the finger cuff, which were sensed by pressure transducer and transmitted to and recorded by the EndoPAT 2000 device. Blood pressure (BP) and heart rate (HR) were measured by an automated BP monitor. Endothelial function was assessed via RH-PAT index. An RH protocol consists of a 5 min baseline measurement, after which a blood pressure cuff on the test arm was inflated to 60 mmHg above baseline systolic blood pressure or at least 200 mmHg for 5 min. Occlusion of pulsatile arterial flow was confirmed by the reduction of the PAT tracing to zero. After 5 min, the cuff was deflated, and the PAT tracing was recorded for a further 6 min. The ratio of the PAT signal after cuff release compared with baseline was calculated through a computer algorithm automatically normalizing for baseline signal and indexed to the contra lateral arm. The estimated ratio reflects the RHI. RHI values <1.67 were considered abnormal [20].

### Statistical analysis

Continuous variables are expressed as mean ± standard deviation (SD) or median (if not standard distribution). Wilcoxon signed-rank test was used to assess the differences between baseline and final characteristics of the study cohort. Categorical variables were compared by Chi square test or Chi square test with Yate’s correction. Spearman’s correlation was used to evaluate the association between metabolic risk factors, RHI and biomarkers. The correlations were adjusted for WC, body mass, BP, lipids and glucose. Mann–Whitney test was used to compare historical LTPA during different life periods. Logarithmic transformation of skewed data did not change the direction or strength of the analyzed calculations. A p value <0.05 was considered statistically significant. All analyses were performed with STATISTICA Windows XP version 9.1.

## Results

Table 1 presents 25-year changes in lifestyle and clinical characteristics of the studied group between the baseline and final examination. While most of the parameters worsened, the number of regular smokers fell and HDL-C level increased substantially throughout the observation (p < 0.05). The studied population is thought as metabolically healthy at baseline as only two persons had more than one MetS components (borderline values of systolic blood pressure and triglycerides).

**Table 1:** Baseline and final characteristics of the studied group (n = 101)

	Baseline	Follow-up
Age, years	35.3 ± 6.4	59.7 ± 9.0***
Waist circumference, cm	85.1 ± 6.9	93.4 ± 7.9*
BMI, kg/m^2^	24.3 ± 2.8	25.1 ± 3.2
Body fat, %	16.1 ± 4.2	20.2 ± 5.8*
Waist circumference ≥102 cm	1	24**
Systolic blood pressure, mmHg	120.3 ± 12.9	128.4 ± 12.7*
Diastolic blood pressure, mmHg	78.1 ± 6.6	79.6 ± 7.7
Blood pressure ≥130/85 mmHg, n	19	42^*^
Triglycerides, mmol/l	1.27 ± 0.44	1.19 ± 0.44
TG ≥1.7 mmol/l, n	16	15
HDL-C mmol/l	1.30 ± 0.28	1.57 ± 0.28*
HDL-C <1.0 mmol/l	8	6
Fasting plasma glucose, mmol/l	4.52 ± 0.56	4.99 ± 0.46*
Fasting plasma glucose ≥6.1 mmol/l, n	1	12**
Uric acid, µmol/L	0.09 ± 0.01	0.10 ± 0.02
Metabolic syndrome, n	0	9**
TG/HDL ratio	0.98 ± 0.4	0.77 ± 0.3
Current smokers, n	17	5*
Alcohol consumption, units/week	4.71 ± 3.6	5.63 ± 4.3
Leisure-time physical activity, hours/week		
Low/moderate intensity (≤6 METs)	2.9 ± 2.98	3.0 ± 2.66
High/very high intensity (>6 METs)	4.8 ± 1.9	4.1 ± 1.1
Exercise-related energy expenditure, kcal/week	3101.3 ± 2871.8	2735.3 ± 1872.5
PWC, W/kg	2.41 ± 0.56	1.97 ± 0.49*

Data presented as mean ± SD unless otherwise stated; * p < 0.05; ** p < 0.01; *** p < 0.001

*BMI* body mass index, *HDL-C* high density lipoproteins, *PWC* physical working capacity

Among analyzed biomarkers, hsCRP, ox-LDL, Il-6, leptin and adiponectin/leptin ratio were significantly related with traditional metabolic risk factors (Table 2). Waist circumference was positively related with hsCRP (p < 0.01), ox-LDL (p < 0.05), leptin (p < 0.05) and negatively with adiponectin/leptin ratio; HDL-C was negatively related with hsCRP (p < 0.01), Il-6 (p < 0.05) and positively with adiponectin/leptin ratio (p < 0.05); TG level was positively related to leptin (p < 0.01) and negatively to adiponectin/leptin ratio (p < 0.05); uric acid was positively related with hsCRP (p < 0.05), leptin (p < 0.01) and negatively with adiponectin/leptin ratio (p < 0.05). Significant negative correlations was found between RHI and sICAM-1 (p < 0.05) and Il-6 (p < 0.05).

**Table 2:** Correlation of traditional metabolic risk factors and reactive hyperemia index to novel CVD biomarkers at follow-up

	WC	HDL-C	SBP	TG	glucose	Uric acid	Reactive hyperemia index
hsCRP, mg/L	0.255**	−0.272**	0.158	0.151	0.068	0.199*	−0.158
Homocysteine, µmol/L	−0.068	−0.038	0.183	0.049	−0.024	0.058	0.017
ox-LDL, ng/mL	0.230*	−0.156	0.171	0.151	0.173	0.106	0.018
sICAM-1, ng/mL	0.111	0.045	0.041	0.053	−0.122	0.161	−0.228*
sVCAM-1, ng/mL	0.095	−0.008	−0.035	−0.011	−0.127	0.039	−0.070
Interleukin-6, pg/mL	0.059	−0.215*	0.157	0.166	0.043	0.075	−0.257*
Leptin, ng/mL	0.193*	−0.171	0.107	0.282**	0.043	0.285**	−0.010
Resistin, ng/mL	−0.025	0.067	0.148	0.021	−0.087	0.027	−0.145
Adiponectin, µmol/L	−0.051	−0.132	−0.148	0.051	−0.089	0.077	0.102
Adiponectin/leptin ratio	−0.177*	0.193*	−0.158	−0.209*	−0.045	−0.222*	0.060
Irisin, µmol/L	0.005	−0.090	−0.124	−0.015	0.017	−0.042	−0.039

*WC *waist circumference, *SBP* systolic blood pressure, *TG* triglycerides, *hsCRP* high-sensitvity C-reactive protein, *sICAM-1* soluble intracellular adhesion molecule-1, *sVCAM-1* soluble vascular cell adhesion molecule-1, *ox-LDL* oxidized low-density lipoproteins

* p < 0.05; ** p < 0.01; *** p < 0.001

Table 3 shows relationships between metabolic parameters, LTPA characteristics and endothelial function in the whole group. Significant positive correlations were found between RHI, HDL-C (p < 0.01), current and historical LTPA (p < 0.01). Body mass, WC, TG, TG/HDL ratio, uric acid and number of metabolic risk factors were negatively related to RHI.

**Table 3:** Correlation between metabolic risk factors and reactive hyperemia index at final examination

Metabolic risk factors	Reactive hyperemia index
Waist circumference, cm	−0.18*
Body mass, kg	−0.26**
Systolic blood pressure, mmHg	−0.13
Diastolic blood pressure, mmHg	−0.08
Triglycerides, mmo/l	−0.22**
HDL-C, mmol/l	0.25**
TG/HDL ratio	−0.24**
Glucose, mmol/l	−0.02
Uric acid, µmol/L	−0.21*
Current LTPA, kcal/week	0.27**
Historical LTPA since 12th year of life, hours/year	0.29**

Data presented as mean ± SD unless otherwise stated; * p < 0.05; ** p < 0.01; *** p < 0.001

*HDL-C* high density lipoproteins, *LTPA* leisure-time physical activity

Approximately half of the studied cohort remained metabolically healthy at follow-up (Table 4). At baseline no significant differences in traditional CVD risk factors were found between metabolically healthy and unhealthy groups. At follow-up metabolically unhealthy individuals had significantly higher percentage of body fat (p < 0.001), higher hsCRP and uric acid (p < 0.05) and lower adiponectin/leptin ratio (p < 0.05). They had also significantly higher mean values of triglycerides (1.30 vs 1.03 mmol/l; p < 0.01) and glucose (5.23 vs 4.75 mmol/l; p < 0.01), and lower HDL-C (1.40 vs 1.77 mmol/l; p < 0.01) as compared to metabolically healthy subjects.

**Table 4:** Anthropometric, biochemical and endothelial indices according to metabolic status at follow up

	Metabolic status at follow-up	
	Metabolically healthy (0–1 factors) n = 47	Metabolically unhealthy (≥2 factors) n = 54
Age, years	61.0 ± 10.1	59.1 ± 8.4
Body fat, %	17.1 ± 4.0	22.2 ± 4.7***
Waist circumference ≥102 cm, n	1	23***
BMI ≥30 kg/m^2^, n	0	8*
RHI	2.06 ± 0.48	1.91 ± 0.42*
RHI <1.67, n	5	15*
Uric acid, µmol/L	0.08 ± 0.01	0.10 ± 0.02*
hsCRP, mg/L	2.31 ± 1.15	2.73 ± 1.14*
Homocysteine, µmol/lL	13.25 ± 6.3	14.95 ± 4.7
ox-LDL (median), ng/mL	99.85 ± 181.4 (29.1)	115.92 ± 226.7 (39.5)
sICAM-1, ng/mL	486.60 ± 117	502.25 ± 143.53
sVCAM-1, ng/mL	657.58 ± 250.0	749.05 ± 263.4
Interleukin-6, pg/mL	2.67 ± 2.1	2.78 ± 2,7
Leptin, ng/mL	5.91 ± 4.5	7.75 ± 6.4^#^
Resistin, ng/mL	4.52 ± 1.8	4.99 ± 2.9
Adiponectin, µmol/L	8.15 ± 3.2	7.48 ± 2.5
Adiponectin/leptin ratio	1.41 ± 0.7	0.91 ± 0.4*
Irisin, µmol/L	0.49 ± 0.2	0.50 ± 0.1
EE at baseline, kcal/week	4558.7 ± 3233.0	2638.5 ± 2198.7***
EE at follow-up, kcal/week	3230.8 ± 2150.0	2661.0 ± 2375.6*
VO_2_max, ml/kg/min	35.1 ± 8.9	30.1 ± 8.2*
PWC, W/kg	2.09	1.83*
Smoking at baseline, n	9	8
Smoking at follow-up, n	3	2

Abbreviations as in the previous tables

* p < 0.05; ** p < 0.01; *** p < 0.001; ^#^p = 0.0502

Regarding endothelial function, metabolically healthy individuals had higher mean RHI and lower number of decreased RHI (p < 0.05) (Table 4). Metabolically healthy individuals had significantly higher LTPA level and aerobic capacity at baseline and follow-up. Of 15 smokers at baseline, nine individuals followed the medical advice and gave up smoking during first 3 years of the observation. Only one person gave up smoking 5 years before the latest follow-up. No significant difference were found in terms of smoking history and alcohol consumption between the groups (Table 4).

Metabolic syndrome was found in 9 individuals at follow-up. Among metabolically unhealthy subjects (n = 51) no substantial differences in biomarkers profile and RHI were noted between those with and without MetS (data not shown in the tables).

## Discussion

To our knowledge this is the first research on the relationship between long-term changes of metabolic status, novel CVD biomarkers and microvascular endothelial function in the cohort of men tracked prospectively for about 25 years.

According to our results leptin, adiponectin/leptin ratio and hs-CRP are significant predictors of metabolic status. Most traditional risk factors, uric acid, Il-6 and sICAM-1 correlate with endothelial function as measured by RHI. Particularly important finding of this analysis is that even subtle changes in metabolic profile in asymptomatic physically active individuals modify inflammatory markers and microvascular endothelial function. Maintaining metabolically healthy status through middle adulthood is associated with better endothelial function and more favorable biochemical profile. Among the analyzed lifestyle-related factors, long-term physical activity level occurred the most important contributor of metabolic changes in the studied cohort.

Metabolic syndrome is a constellation of factors deeply involved in atherosclerosis. Even in asymptomatic individuals, presence of larger WC, elevated TG, low HDL-C, prediabetes or prehypertension may accelerate development of atherosclerosis [21, 22]. Impaired endothelial function is among the earliest detectable disturbance in the natural history of atherosclerosis [23]. Li et al. showed that individuals with MetS have a higher degree of endothelial dysfunction compared with individuals with multiple CV risk factors [24]. These findings emphasize a unique position of metabolic disturbances in prediction of adverse CV events beyond the contributions of traditional risk factors. It seems that factors which are not commonly included into popular risk algorithms (as visceral adiposity, body weight, glucose profile) provide substantial information on vascular health. Endothelial dysfunction was also found among asymptomatic individuals with obesity and prediabetes. Gupta et al. demonstrated that obese men and women with predisease conditions had resting endothelial dysfunction as compared to non-obese persons with normal glucose and blood pressure [25]. Also weight gain in healthy individuals may result in cardiovascular risk escalation, including impaired endothelial function [26]. In our study we did not find substantial differences in endothelial function between individuals with and without MetS. It was expected due to a small number of men who developed MetS during the observation (n = 9). However, significantly better RHI values (both mean and number of subjects with RHI <1.67) were observed among those who maintained metabolically healthy profile. These findings suggest that even subtle changes in metabolic parameters among asymptomatic individuals may accelerate endothelial dysfunction.

Most, but not all, metabolic components occurred significantly correlated with endothelial function which is in line with some previous studies in this field [27–30]. We have not found, however, independent correlation between FPG, blood pressure and endothelial function which is consistent with some other studies that incorporated reactive hyperemia index measurement [24, 29]. In contrast, Carnovale et al. reported strong relationship between all MetS components and RHI, but all the participants of this study (including post-menopausal women) had central obesity and differed substantially from our cohort [30].

Precise mechanism linking metabolic disorders with endothelial dysfunction are not clear. Apart from the traditionally measured parameters, insulin resistance is thought to exert specific role in vascular health. Insulin resistance may impair nitric oxide bioavailability and itself cause endothelial damage [31]. In the present analysis, we have not measured insulin resistance. However, substantial abnormalities in insulin metabolism would be improbable due to a relatively high PA level of our cohort. Some previous studies and the latest report of Peterson et al. indicate that moderate and vigorous PA is among the strongest predictors of insulin resistance in middle-aged and older men [32].

Besides insulin resistance, several biochemical markers are involved in endothelial homeostasis, metabolic risk and atherosclerosis. Recent findings from basic science highlight the fundamental role of inflammation in all stages of development of atherosclerosis. The strongest evidence is associated with hsCRP [33]. Several studies demonstrated independent association between elevated hsCRP levels and increased CVD risk as well as impairment of insulin signaling and reduced nitric oxide release from endothelial cells [33–36]. Elevation of hsCRP indicating a chronic low-grade inflammation predicts risk of atherosclerotic complications independently of traditional CVD risk factors and myocardial damage [33]. Our results showed significant relationship of hsCRP with WC, HDL-C and uric acid. Importantly, metabolically healthy individuals had significantly lower levels of hsCRP as compared to those who developed at least two metabolic risk factors. This finding is of special importance as individuals with both increased inflammation and endothelial dysfunction represent particularly high CVD risk [3]. However, no significant correlation was found between hsCRP and RHI. Similar results were reported by Konttinen et al. which might suggest that other biomarkers would be more sensitive in identifying the earliest stages of endothelial dysfunction [29].

Several studies have demonstrated that increased level of serum uric acid, even within the normal range, is associated with CVD risk factors and future development of MetS [37, 38]. This is in line with our findings as uric acid occurred significantly higher in metabolically unhealthy individuals at follow up. We are unaware of any study assessing the relationship between uric acid and reactive hyperemia index in asymptomatic adults using a peripheral arterial tonometry device. We found that serum uric acid was significantly related to RHI which is consistent with some other studies measuring endothelial function with different methods [39]. This finding emphasize the position of this marker as an independent predictor of metabolic disorders and endothelial dysfunction.

However, it should be noted that inflammatory markers do not pinpoint the exact location of inflammation. C-reactive protein and some other biomarkers measure general levels of inflammation and elevated levels may be caused by infections and many chronic conditions. In order to find the exact site of tissue inflammation more specific test may be performed. In the context of vascular and cardiometabolic health, adipose tissue seem particularly important. There is a growing evidence that adipose tissue is an active organ able to release several mediators affecting metabolic and vascular health like Il-6, TNF-alpha, adipokines [33, 40]. Excess adipose tissue is accompanied macrophage infiltration and cytokines released in this process spill into the circulation and probably result in systemic inflammation. Elevated CRP level seem to reflect a systemic response to localized inflammation in adipose tissue [41].

Among adipocyte-derived factors, the principal role in modifying metabolic profile has been established for leptin and adiponectin [42–45]. Leptin, an adipocyte-derived hormone, is involved in angiogenesis, oxidative stress, sympathetic activation and prothrombotic processes. Majority of studies have documented a significant relationship between higher leptin concentrations and an increased risk of obesity, hypertension, metabolic syndrome as well as markers of inflammation and thrombophilia [42–45]. Serum leptin has also been identified as an independent predictor of metabolic syndrome in men [46]. In contrast to leptin, adiponectin, an adipocyte-derived collagen-like protein, has been generally reported as a protective factor with an anti-inflammatory and anti-atherogenic properties [47].

Consistently to some previous findings, our results indicated that leptin was positively associated with waist circumference, TG and uric acid [43, 46]. Furthermore, we observed that adiponectin/leptin ratio was significantly higher in metabolically healthy men. Significant correlation between adiponectin/leptin ratio and TG confirms the results obtained by Vega and Grundy [48].

Due to the rising interest in the role of irisin in prevention of obesity and metabolic disorders, we included this novel myokine into the analyzes. Irisin has been shown to induce conversion of white adipocytes into brown-like adipocytes [49]. To date, different researchers have reported conflicting results [49–51]. In our study we have not found any significant associations between irisin and analyzed parameters. Similarly, Huh et al. have recently found that the exercise-induced change in irisin levels did not differ between healthy individuals and subjects with metabolic syndrome regardless of exercise type [50]. However, in another study, circulating irisin was negatively correlated with BMI and waist circumference and positively with endothelial function [51]. These discrepancies may result from substantial methodological differences between the studies. Our cohort was much older, with various metabolic disorders while the population of the study of Hou et al. (n = 41) consisted of nonhypertensive, not smoking, nondiabetic obese and lean healthy controls. Moreover, endothelial function was measured by means of ultrasound scanner. Further studies with larger sample sizes and more severe metabolic disorders are needed to clarify the role of irisin in modifying metabolic profile and endothelial function.

Reactive hyperemia index occurred inversely associated with sICAM-1 and Il-6 which is in line with the findings obtained by other authors investigating the association between inflammatory biomarkers and endothelial function [30, 52–54]. The initial stages of atherosclerosis associated with adhesion of circulating leukocytes to the endothelial cells are mediated by cellular adhesion molecules, including sICAM-1 [55]. Interleukine-6 is also thought as a key inflammatory cytokine that plays a central role in propagating the downstream inflammatory response responsible for atherosclerosis [56]. Both markers reflect impaired microvascular function and could be, therefore, used as early markers of endothelial dysfunction.

It would be worthwhile to speculate why some of the participants developed metabolic disorders while others remained metabolically healthy. Among possible contributors we considered several factors, including smoking (especially smoking cessation), alcohol consumption, diet and physical activity level. Baseline and final characteristics of metabolically healthy and unhealthy individuals revealed substantial differences only in terms of weekly exercise-related energy expenditure and aerobic capacity. Therefore, it seems that long-term PA pattern was the most important contributor of metabolic changes in the studied cohort.

The obtained results support our previous findings that sufficient prevention of metabolic disorders may be achieved with regular high PA level substantially exceeding current recommendations [12]. Importantly, in the metabolically healthy group percent of body fat remained stable throughout the whole observation and only one person developed central obesity. The above effects together with better endothelial function may be associated with dose and intensity of exercises. Interesting study of Hallmark et al. revealed that high-intensity exercises acutely enhanced endothelial function, while moderate-intensity exercise was not significant in young lean subjects. The endothelial response of obese adults was blunted compared to lean adults [57]. Of note, vast majority of metabolically healthy subjects in our study was involved mainly in vigorous exercises.

A number of limitations of the present study should be acknowledged. The study is not representative for middle-aged men, because the participants were not randomly selected from the general population. Well known limitation is related to self-reported questionnaires on PA which are prone to recall bias. Due to the cross-sectional assessment of novel biomarkers and indices of endothelial function only at follow-up examination we could not fully determine the direction of causality. Unfortunately we did not assess free fatty acids in our cohort. However, owing the significance of this factor in metabolic characteristic of the MetS, we will include free fatty acids into the panel of measured markers during next follow-up.

Among various methods to measure endothelial function, we chose the system assessing microvascular endothelial function. The endoPAT system is a relatively simple, non-invasive, digital method of assessing microvascular function by measuring postocclusive reactive hyperemia. Another recognized non-invasive method of measuring endothelial function is brachial artery flow-mediated dilation (FMD) which is based on the measurement of the brachial artery diameter before and after acute brachial artery occlusion [58]. However, FMD is technically challenging, relatively complex, and the results are difficult to compare between clinical laboratories. Direct comparison of both methods is difficult as the brachial and digital measures of vascular function provide distinct information and therefore may reflect different conditions. Growing evidence show that RH-PAT may be complementary to FMD. Importantly, Sauder et al. have recently demonstrated that peripheral arterial tonometry can be used to assess endothelial dysfunction in adults with the metabolic syndrome as reliably as in healthy samples [59]. Finally, it should be noted that the experimental design of the study did not entail vascular smooth muscle function.

The distinct strength of the study is a long-term prospective observation of CVD risk factors and health-related behaviors in a unique cohort of men with comparable metabolic profile at baseline. We assessed a large set of biochemical markers in relation to traditional metabolic risk factors and reactive hyperemia. Longitudinal observation of a homogenous group enables to eliminate the risk associated with such known confounders such as age, social class or lifestyle choices. We excluded individuals taking drugs modifying CVD risk, which could influence on endothelial function in order to reduce the confounding effect of anti-atherogenic treatment.

In summary, this prospective study in physically active asymptomatic men indicated that even subtle changes in metabolic profile may have an impact on traditional and novel CVD biomarkers as well as microvascular endothelial function. Maintaining metabolically healthy status through middle adulthood may protect against endothelial dysfunction. Leptin, adiponectin/leptin ratio and hsCRP are significant predictors of metabolic profile. Among analyzed biomarkers, Il-6 and sICAM-1 may be used as indicators of an early endothelial dysfunction. Long-term high PA level accompanied by high aerobic capacity are principal lifestyle-related contributors of metabolically healthy profile.
